# "Harshlighting" small blemishes on microarrays

**DOI:** 10.1186/1471-2105-6-65

**Published:** 2005-03-22

**Authors:** Mayte Suárez-Fariñas, Asifa Haider, Knut M Wittkowski

**Affiliations:** 1 Center for Studies in Physics and Biology, The Rockefeller University, 1230 York Ave, Box 212, New York, NY 10021, USA; 2 Laboratory of Investigative Dermatology, The Rockefeller University, 1230 York Ave, Box 178, New York, NY 10021, USA; 3 General Clinical Research Center, The Rockefeller University, 1230 York Ave, Box 322, New York, NY 10021, USA

## Abstract

**Background:**

Microscopists are familiar with many blemishes that fluorescence images can have due to dust and debris, glass flaws, uneven distribution of fluids or surface coatings, etc. Microarray scans show similar artefacts, which affect the analysis, particularly when one tries to detect subtle changes. However, most blemishes are hard to find by the unaided eye, particularly in high-density oligonucleotide arrays (HDONAs).

**Results:**

We present a method that harnesses the statistical power provided by having several HDONAs available, which are obtained under similar conditions except for the experimental factor. This method "harshlights" blemishes and renders them evident. We find empirically that about 25% of our chips are blemished, and we analyze the impact of masking them on screening for differentially expressed genes.

**Conclusion:**

Experiments attempting to assess subtle expression changes should be carefully screened for blemishes on the chips. The proposed method provides investigators with a novel robust approach to improve the sensitivity of microarray analyses. By utilizing topological information to identify and mask blemishes prior to model based analyses, the method prevents artefacts from confounding the process of background correction, normalization, and summarization.

## Background

Analysis of hybridized microarrays starts with scanning the fluorescent image. For high-density oligonucleotide arrays (HDONAs) such as Affymetrix GeneChip^® ^oligonucleotide (Affy) arrays, the focus of this paper, each scanned image is stored pixel-by-pixel in a 'DAT' file. As the first step in measuring intensity of the hybridization signal, a grid is overlaid, the image is segmented into spots or features, and the pixel intensities within each of these are summarized as a probe intensity estimate (See reviews [1] and [2] for cDNA chips). The probe-level intensity estimates are stored in a 'CEL' file. Each gene is represented by pairs of probes, each representing another characteristic sequences and a 'mismatch', which is identical, except for the Watson-Crick complement in the center. Expression of a gene is estimated from such a probe set by applying algorithms for background correction, normalization, and summarization.

The quality of data scanned from a microarray is affected by a plethora of potential confounders, which may act during printing/manufacturing, hybridization, washing, and reading. Each chip contains a number of probes specifically designed to assess the overall quality of the biochemistry, such as 'checkerboards' in the corners and borders, whose purpose is, e.g., to indicate problems with the biotinylated B2 hybridization. Affymetrix software provides for a number of criteria to assess the overall quality of a chip, such as percent present calls, scaling factor, background intensity, and overall pixel-to-pixel variation (raw Q). Software packages such as Bioconductor for R [3] have implemented biochemical quality control tools such as RNA degradation plots. If a quality problem is found, however, these criteria and tools do not easily suggest a remedy and they have little sensitivity to detect localized artefacts, like a speck of dust or a localized hybridization problem. Although such physical blemishes obviously affect the expression estimates, they have hitherto been only narrowly addressed in the literature. Thus, there are currently no safeguards to signal potential physical blemishes. Instead, researchers are merely advised to carefully inspect the chip images visually [4,5]. Given the high variance among the hundreds of thousands of probes and their random allocation on the chip, it is impossible to visually detect any but the starkest artefacts. For two-colour cDNA arrays, a Bayesian network approach has been proposed [6], based on the 'features' of the pixel distribution within each probe, yet, due to the standardized manufacturing process, the probes on an oligonucleotide array have too few 'features' for such an approach to be effective.

As the price of microarrays continues to drop, a typical microarray experiment now contains several chips, each representing a sample obtained under conditions that were similar except for the experimental factor under investigation. Having collections of chips available offers new strategies not only for analyzing the effect of the experimental factor, but also for identifying blemishes. The power of having several chips available was first harnessed for estimating mRNA expression levels by the 'robust multichip average' (RMA) method [7]. One of the assumptions underlying the RMA model is that probes across chips are highly correlated, due to differences in their affinity [8,9] and because only a small proportion of genes are differentially expressed in any experimental setting. This correlation should be even higher for the mismatches, because they are less likely to be affected by the specific changes in gene expression induced by the experimental factor. Given the volume of pixel level data, (>50 megapixels per image) it is desirable to devise algorithms that work from the 100 times smaller probe level files, the same information used in traditional signal value estimation approaches.

**Figure 1:**
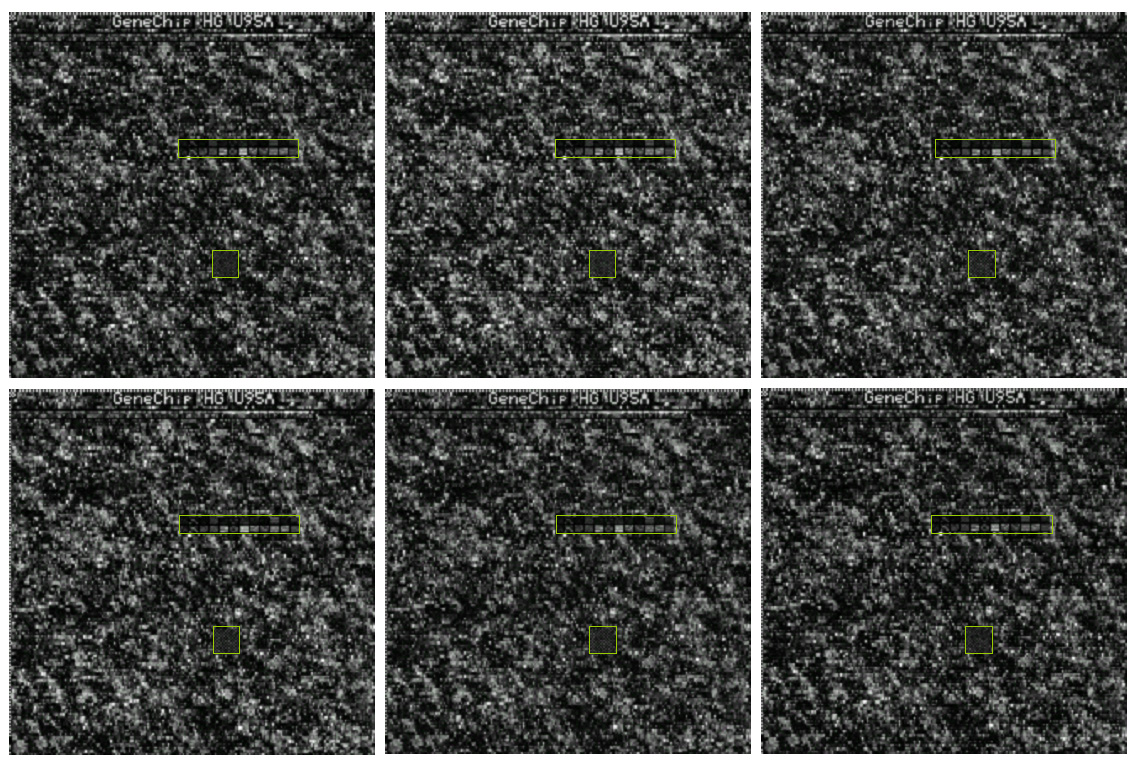
**Detail of six chips for the second patient**. Upper left corners of six chips (250 × 250 cells) with samples from a cell culture evaluated at different time points (rows: 6 hrs, 24 hrs) under different experimental conditions (columns: none, activated, activated in the presence of a repressor). Boxed areas indicate internal standards.

Figure 1 shows how the large probe-to-probe variance can obscure the subtle changes caused by all but the starkest blemishes. Since probes vary in intensity by four orders of magnitude, a biologically relevant change of 30% in brightness in a small region can easily go undetected. In fact one of the chips shown in Figure 1 is affected by several blemishes. However, Figure 1 shows that not only do the internal standards have a very consistent pattern, as one would expect, but also that high expression values are correlated across chips. As we will show, drawing on these correlations allows for a simple and efficient method to identify areas on individual chips where the assumption of spatially uncorrelated errors is clearly violated. We shall use the chip-to-chip correlation to devise a 'harshlight' that makes the blemishes stand out.

## Results

### Data

Psoriasis is thought to be due to an overly active immune system [10,11]. To study how the immune response of leukocytes isolated from blood can be affected by drugs that may serve to control autoimmune diseases like psoriasis, blood was drawn from five volunteers under a protocol that had been approved by The Rockefeller University Hospital Institutional Review Board [12].

For each subject, peripheral blood mononuclear cells (PBMCs) were isolated and cultured in six Petri dishes. Four cultures were activated with an anti CD3/CD28 antibody, two of which were pre-treated with a repressor drug. Two cultures served as control without drug or activation. One of the two sets of control, activated, and pre-treated cultures (subject 1 and 2) was analyzed after 6, the other after 24 hrs. (For subjects 3, 4, and 5, only one time point is available.) All samples were hybridized to Affymetrix HuU95av2 chips.

### Artefacts identified on probe-level (CEL) files

**Figure 2:**
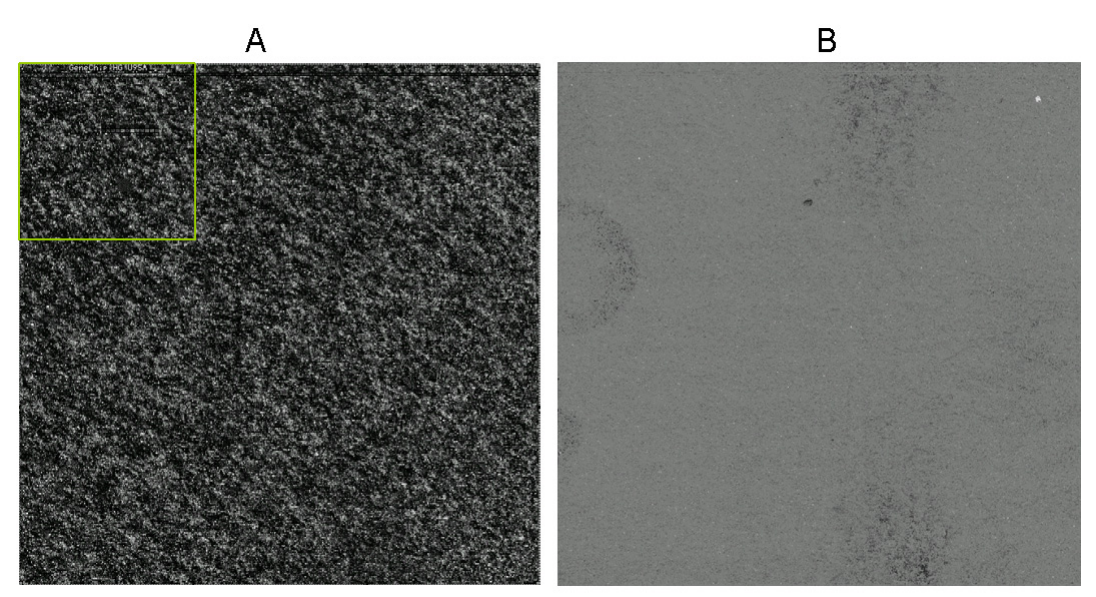
**Blemishes of one chip (Activated 6 h) for the second patient**. Left (a), whole chip pseudo-image. The box indicates the portion of this chip depicted in the center image of the first row of Figure 1. Right (b), filtered image based on the set of the three chips in the first row (with median adjustment).

Figure 1 displays the six chips obtained from one subject's PBMC sample. This subject was chosen because one of the chips (upper row, centre) exhibits a variety of blemishes, which are discussed below, see Figure 2b: a 'bright spot' in the upper-right corner, a 'dark spot' in the upper centre, 'dark clouds' in the upper and lower right centre, and two 'shadowy circles' reaching beyond the left border. Part of the upper circle is included in the chip portion depicted in Figure 1.

**Figure 3:**
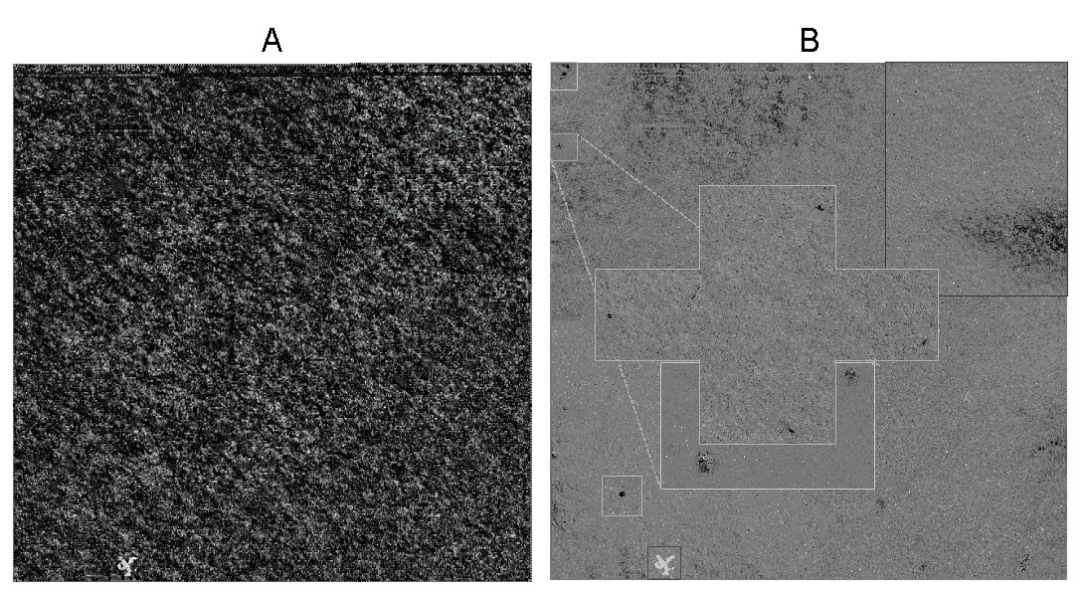
**Collage of Artefacts**. b) Areas with artefacts obtained from seven chips. a) Composition of the raw areas corresponding to the areas denoted in (b). Filtered image based on the set of three chips with median adjustment.

Similar results were obtained for all subjects (data not shown). None of the artefacts would have been detected by visual inspection of the pseudo image (Figure 2a). Even after having seen the filtered image, most blemishes are difficult to identify at best. Interestingly, some chips appear to have a preponderance of specific artefacts, suggesting that at least some of the blemishes are caused by specific environmental factors during hybridization, and providing the first indication for the validity of the proposed method. The chip used as the background in Figure 3 has 'dark clouds' in the upper left corner and, albeit to a lesser degree, in both lower corners. Of the two chips with several smaller artefacts, one had three spots that resemble the 'dark spot' in Figure 2. Only the bright scratch at the bottom of one of the chips could have been detected by mere visual inspection of the chip, although even this chip passed the Rockefeller University's Gene Array Resource Center's quality control.

### Average *vs*. median in the filtering procedure

**Figure 4:**
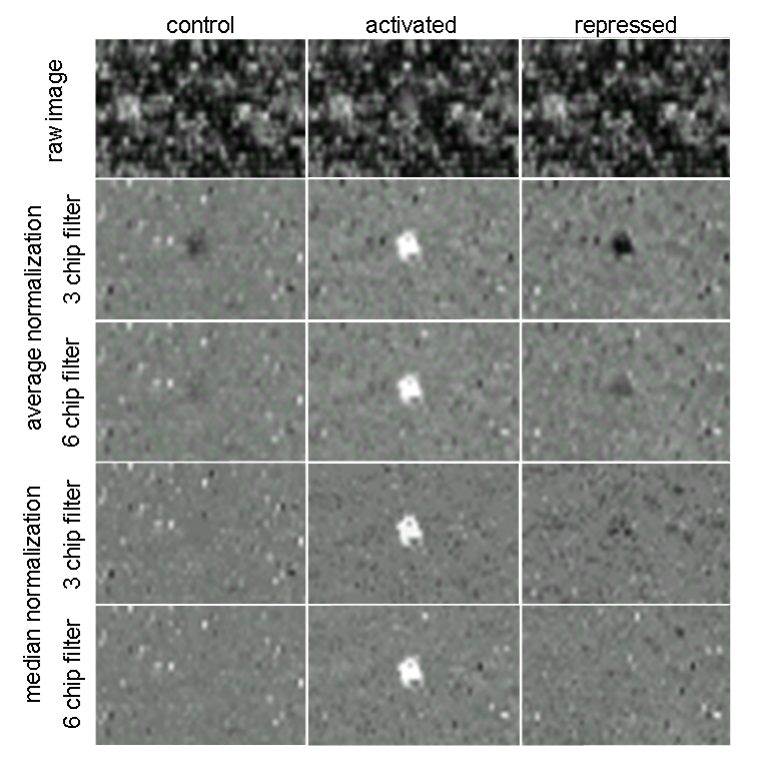
**Median *vs*. Average filter**. The "bright spot" artefact (of Figure 2). Top row: raw image from the same are of three chips showing gene expression from the same sample under three experimental conditions. 3 chip filtering relies on information from the three presented chips measured at 6 hrs only, while 6 chip filtering also draws on the chips observed at 24 hrs.

The proposed filtering process relies on identifying deviations of a probe on one chip from a measure of central tendency for this probe across chips. Thus, if few chips have high intensity 'outliers' for one probe, the chips with normal intensities may appear to be negative 'outliers'. One would expect that the six-chip filter is less likely to generate such 'ghosting' artefacts than the three chip filter. We compared the use of medians *vs*. arithmetic means as the reference. As we had predicted based on the understanding that errors are more likely to be outliers than white noise, using medians not only resulted in less 'ghosting', but also in fewer isolated cells being considered artefacts and, thereby, better contrast (Figure 4).

### Validation of probe-level artefacts by going back to the pixel-level image

Our method allows us to identify spatially correlated regions that are unlikely to originate from random fluctuations. To demonstrate that the statistical anomalies detected in the pseudo images at the probe level (Figure 2 and Figure 3) are, in fact, physical blemishes, we inspected the corresponding raw image at the pixel level. The regular artefacts seen (shadow, circle, cloud, etc.) are clearly blemishes, even if the precise nature of the physical blemish may not be known. Still, the difference in features between blemishes suggests different causes.

A number of factors are known to cause bright or dark spots in fluorescence micrographs. Dust on the front cover slip will cause a dark, out-of-focus shadow. Common white paper is bleached with strongly fluorescent dyes, so fibres from tissue paper ordinarily used for cleaning cause intense glare. Many organic solvents, detergents, and other chemicals will fluoresce when concentrated, so leftover droplets or condensates will appear as bright regions, regardless of whether they are in front or behind the focal plane. A crack in the glass would ordinarily be invisible to fluorescence microscopy – except for its ability to accumulate such substances. Glass will normally be coated with substances to prevent the direct binding of fluorophores to it; however, any damage to the fragile coating will cause fluorescent streaks. Illumination with a coherent source such as a laser, as opposed to a broadband source such as a xenon lamp, has specific artefacts such as speckle. In addition, the arrays themselves are manufactured through photolithographic techniques and may contain occasional damage.

#### Dirt

**Figure 5:**
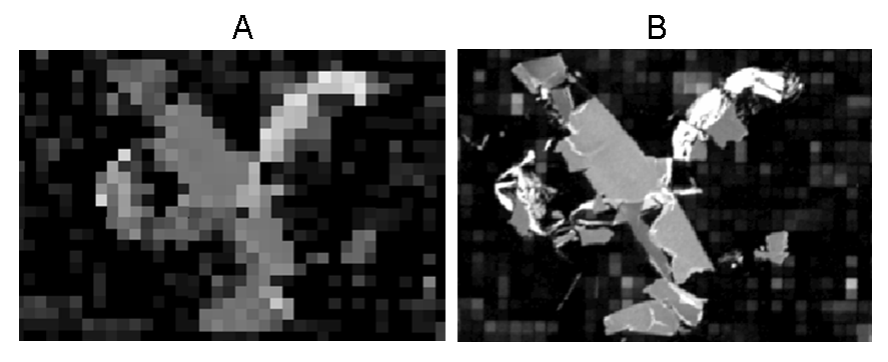
**"Dirt"**. Detail of the artefact seen at the bottom of Figure 3. a) CEL file b) DAT file. The size of this artefact is approximately 25 × 25 probes in the CEL file and 0.5 × 0.5 mm on the chip).

The visible bright artefact at the bottom-left of Figure 3 is the only blemish in our dataset that did not require 'harshlighting' to be visible. The magnification in Figure 5a shows a structure in an area of 25 × 25 probes. Figure 5b shows the corresponding area in the raw image, clearly exhibiting this artefact to be a piece of debris lying in front of the active array surface in the optical path. While the exact physical nature of this debris is unclear, there can be no doubt that probes highlighted at the bottom of Figure 3b are, in fact, a blemish.

#### Dark and bright spots

**Figure 6:**
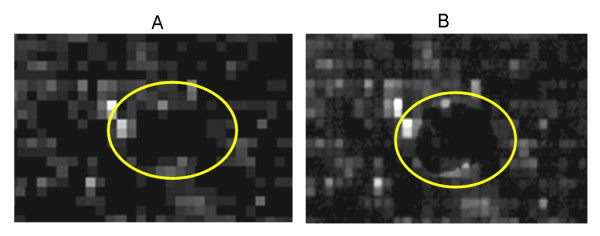
**"Dark Spot"**. Detail of the 'dark spot' artefact seen in the lower left corner of Figure 3. a) CEL file b) DAT file.

A very 'dark spot' was seen in the lower left corner of Figure 3b. The probe level pseudo image (Figure 6a) shows a dark region, but only the raw image reveals the characteristic of this blemish: an elliptical spot with *sharp boundaries *which pass through the inside of probes. Still, the grid is visible underneath, as in one of the examples given by Simon, Korn, et al. [13] for cDNA arrays. The dark probes in Figure 6a are therefore likely to be caused by a physical blemish that has 'stained' the image with a dark oval, a mechanical/optical artefact that invalidates the measured intensities of the probes in the region, so all affected probes in the region should be excluded from further analysis. The 'dark spot' in Figure 2 (upper centre) also had a well defined border, although with less contrast (not shown). Three similar artefacts were seen in yet another chip, as shown in the composite picture (Figure 3).

**Figure 7:**
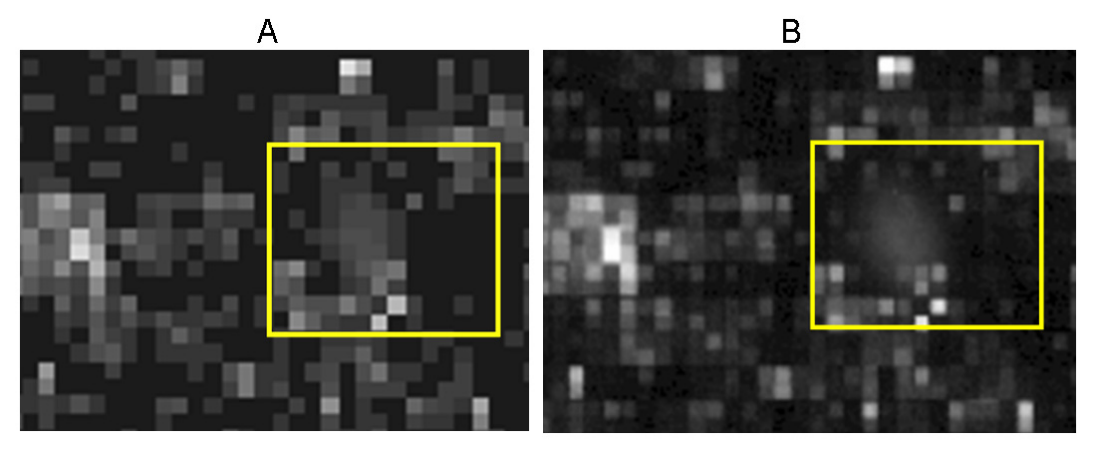
**"Bright Spot"**. Detail of the 'bright spot' in the upper right corner of Figure 3. a) CEL file b) DAT file.

The bright spot on the upper right corner Figure 2 clearly is of different nature. The zoomed area of the DAT file of the second chip (activated) of subject 2 shown in Figure 7b reveals a *diffuse *area of brightness that covers around 20 probes. Because this bright cloud is out of focus, it is difficult to assess whether its physical location was in front of or behind the focal plane; it could be a leftover detergent condensate in the plastic back panel of the chip. The artefact is less visible in the pseudo image than in the raw image, because the low granularity of the pseudo image enforces an artificial grid structure. Moreover, the Affymetrix image analysis algorithm, taking the 75 percentile of the pixels as an estimate of the probe, may make it more difficult to detect these artefacts through visual inspection because the brightness in areas with low pixel-to-pixel variation is lowered for all percentiles above the median. Although they were easily seen in the filtered pseudo image, neither the 'bright spot' nor the 'dark spot' could have been identified by visual inspection of the original pseudo image. Even on the raw image, only an extremely thorough search for areas of low pixel-to-pixel contrast or boundaries with high contrast across probes could have detected these artefacts based on a single chip alone. Thus, blemishes involving only 9 to 25 probes would often be overlooked in a visual inspection of both the raw and the pseudo image. Given the high variance across pixels, any image processing algorithm aiming at detecting such blemishes at high sensitivity would also create many false positive results.

#### Dark clouds

**Figure 8:**
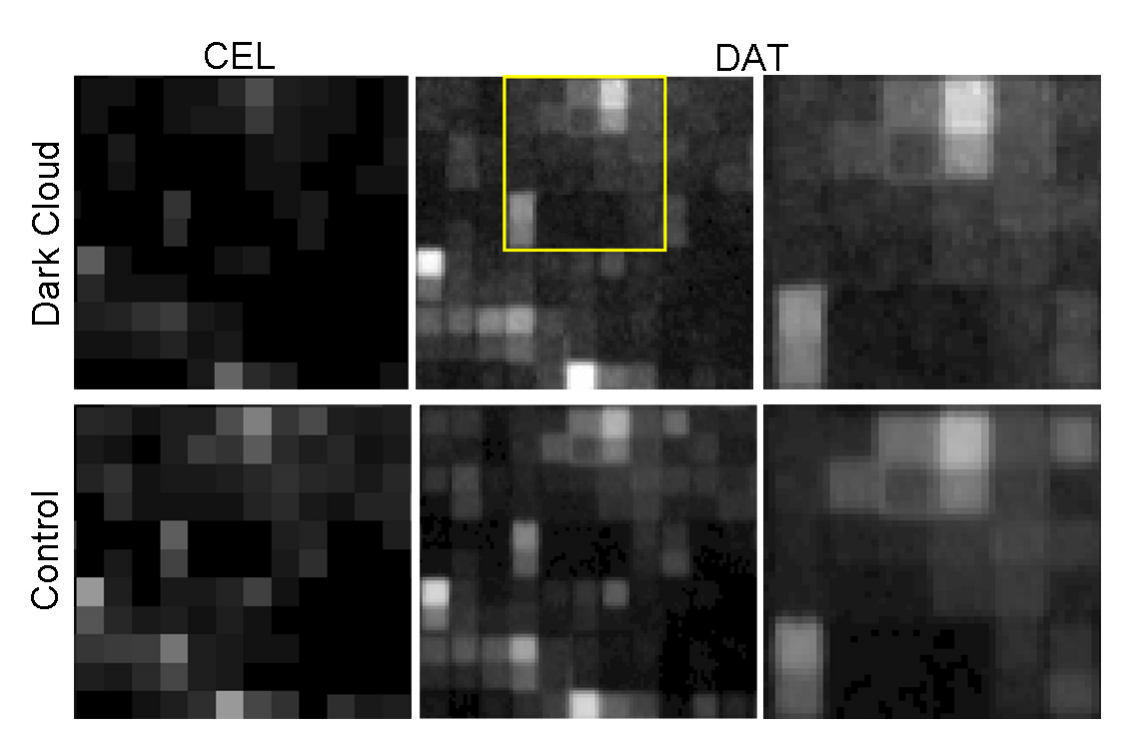
**"Dark Clouds"**. Detail of the region containing the 'dark cloud' at the right border of Figure 1. Top row: sample area around the 'dark cloud', Bottom row: corresponding area from a control chip. 1^st ^column: CEL file, second column: DAT file; third column: detail from the indicated area in the DAT file.

For the 'dark clouds', the raw image at first did not show any recognizable feature. Upon closer inspection, however, we noted that the 'dark cloud' in subject 1 had higher pixel-to-pixel variance (Figure 8). The noise does not seem to have a physical origin, as the fluctuations appear to be single-pixel in extent, giving the raw image a 'grainy' appearance.

The areas outside the dark clouds do not appear to be any grainier, so it does not seem to be a change of exposure setting or other simple global change. The image analysis software reports a single, global pixel-to-pixel variation Q_raw_; it would be useful to have a local quality measure as well, in a fashion similar to the reported background estimate for probe intensities. All dark clouds we found impinge on the array borders. We have no conjecture as to the physical origin of this problem.

#### Shadowy circles

The two artefacts crossing the left border of Figure 2 suggest yet another reason for blemishes on microarrays. Only one of our chips displayed this artefact, but it did so twice on the same border. Neither the raw image nor physical examination of the chip in a dissection microscope provided any hints to the possible cause (data not shown).

There are myriad possible explanations for what caused this striking artefact. A perfectly round structure with outliers concentrated near its perimeter, evocative of the 'coffee stain rings' phenomenon [14], suggests that a bubble (or a drop) may have formed, during the microfluidic stage, condensation after the washing stage, or as a manufacturing defect.

**Figure 9:**
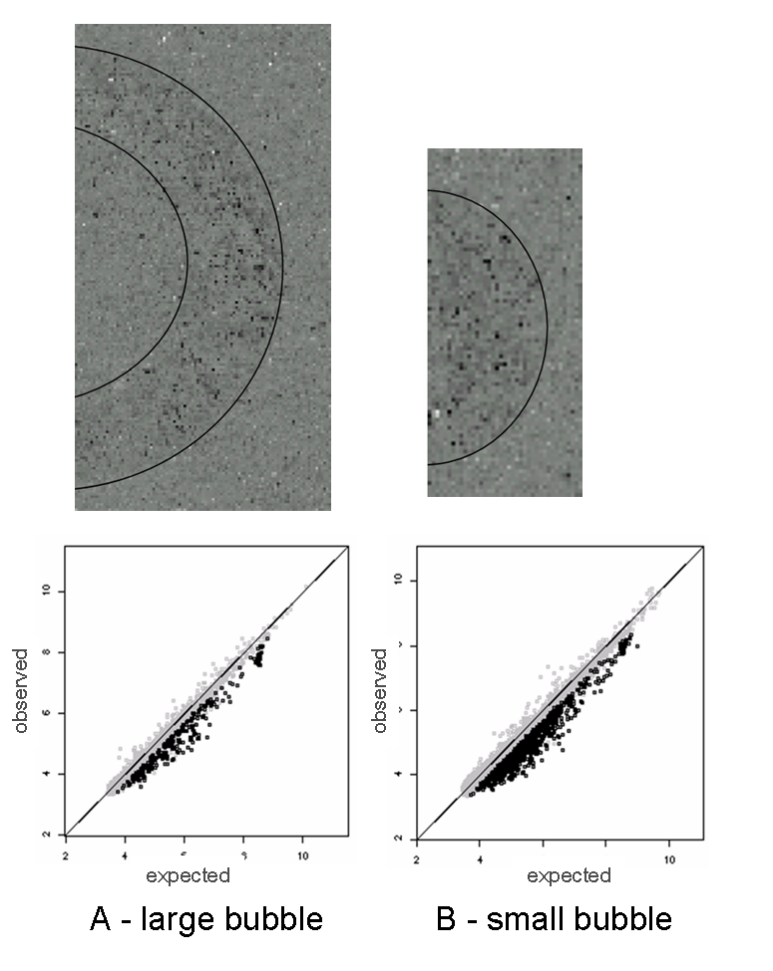
**"Shadowy circles"**. Analysis of the two 'shadowy circles' at the left border of Figure 2. Observed intensities *vs*. expected intensities.

Thus, to further elucidate the potential cause of this artefact, we plotted the observed *vs*. the expected intensity (median across the other five chips) for each probe in the area depicted above (Figure 9). We then marked the points below the .10 percentile of all deviations (3) in this area, which formed the 'shadowy circle'. These points were seen over a wide range in expected intensity (7 to 14 in log_2 _units), although their density is higher for lower intensities. Notably, their intensity was consistently lower than the expected intensity, as though something had only *partially *interfered with hybridization – or partially stripped the fluorophores prior to readout, or affected probe sensitivity.

### Relevance

To determine the extent to which such artefacts may affect standard analyses, we compared the activated *vs*. the repressed samples (two each) for patient 2, and studied whether masking the blemishes affects the list of differentially expressed genes.

**Figure 10:**
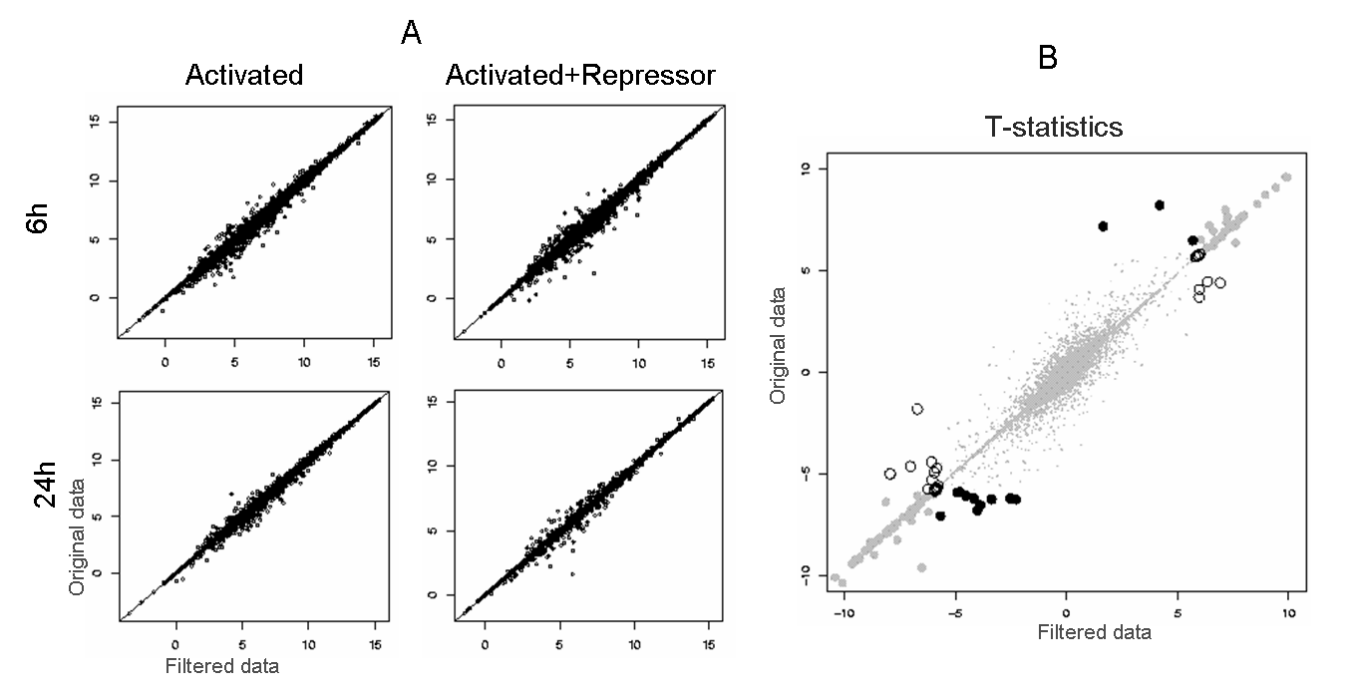
**Influence on expression values**. a) Expression values for four chips of subject 2, original data *vs*. filtered data. b) T-statistics for the comparison of activated *vs*. activated in the presence of the repressor; raw *vs*. filtered data. Open circles: p < 0.01 with the original data only, solid black dots: p < 0.01 with the filtered set only; large grey solid dots: p < 0.01 with both sets.

We searched for blemishes all four chips; after manually circling each affected area, we masked (declared missing) all points in the upper or lower 10th percentile within that area, respectively. We used either the lower or upper 10% since one of our findings is that all artefacts seem to have the common characteristic shown, for instance, in Figure 9, that outliers within an artefact are either (almost) exclusively brighter ('bright spot') or darker (all other blemishes) than expected We conducted separate analyses for the original and the masked data. We estimated the signal value for each probe using the Bioconductor implementation (*affy *package 1.3.28, R.1.8) of the MAS5 algorithm with default parameters, after modifying the summarization and normalization steps to allow for missing data. The overall effect is shown in Figure 10a, with a maximum difference of 4.6 log_2_.

Genes whose expression estimates changed by more than 0.1 log_2 _through filtering were considered as 'altered' by filtering. The 'bright spot', where about 39 probes were affected, altered the expression of 16 genes by up to 1.37 log_2_. The 'shadowy circle' altered the expression of about 380 genes; more than 50 of them by more than 0.5 log_2_. The 'dark spot' affected 47 probes, altering expression of 103 genes by up to 1.6 log_2_. The 'cloud' altered the expression of 700 genes, 83 of them by more than 0.5 log_2_. The dirt covering around 25 × 25 probes, affected around 376 probes, altering 148 genes, 16 of them by more than 0.5 to a maximum of 1.26 log_2_.

Finally, we compared the two conditions (absence *vs*. presence of a repressor), mirroring masked probes on both on the affected and the corresponding chip. As an exploratory criterion, we used the modified (paired) t-test suggested in Smyth [15] from the *limma *package of the Bioconductor project [16]. As shown in Figure 10b, the effects of identifying genes as differentially expressed can be dramatic, demonstrating the potential value of detecting blemishes and masking affected areas on microarrays.

### Validity

**Figure 11:**
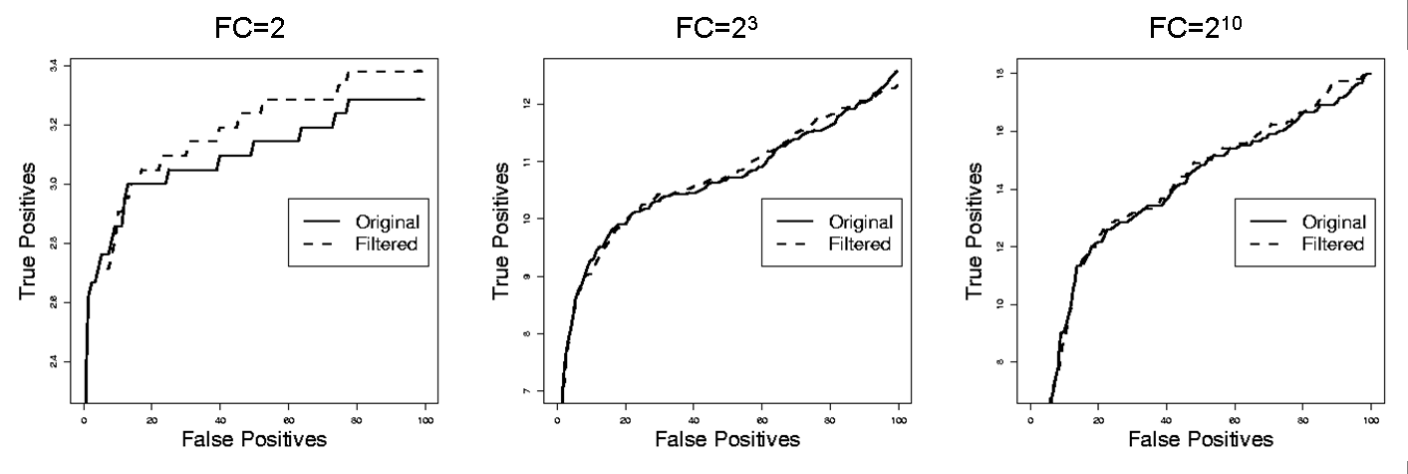
**ROC curves for Spike-in 133 data**. Receiver Operator curves. a) using only arrays which nominal fold changes are equal to 2. b) as a but fold change equal to 8. c) as a but fold change equal to 1024.

We validate the proposed method using data from the Spike-in HUG133 experiments [17]. This data set consists of 3 technical replicates of 14 separate hybridizations of 42 spiked transcripts at concentrations from 0.125 pM to 512 pM arrayed as a Latin Square. Our interest is to assess whether masking the blemishes improves the ability to detect differentially expressed genes. We used the *Affycomp *package of the Bioconductor project, which encompass a series of tools developed by [18] to compare the performance of expression measures for Affymetrix GeneChips. Figure 11 shows that masking blemishes has little effect for large fold changes, as one would expect, while the ROC curve (sensitivity) vs. (1-specificity) shows a substantial improvement for small (2 fold) changes. Other statistics are also improved in this case: the average false positive decreases (from 2818 to2763) while the true positives increases (from 14.33 to 14.57). Comparing by range of intensities, the area under the curve (AUC) is bigger for the masked data in the lower intensities (0.003 *vs*. 0.010) while keep similar performance in the medium and low range (data not shown), resulting in a bigger average weighted AUC for the filtered data (0.002 *vs*. 0.007) (a detailed description of these statistics can be found in [18] and in the *affycomp *vignette). Thus, our masking procedure improves the sensitivity/specificity to detect small differential expression, especially in the range of low intensities.

## Discussion

As an alternative approach to identify blemishes, one might try to look at the residuals from parametric estimations in the background subtraction or summarization stages; e.g., looking at the residuals of the PM-MM difference model [19] or the RMA model over the PM values [20] to identify possible aberrations. Unfortunately, the variety of models currently being discussed attests to the fact that each model has its drawbacks. While random variation can typically be handled by statistical methods, systematic errors in the choice of the model assumptions may have a drastic impact on these processes. The proposed method is robust in the sense that only few assumptions are made. Another advantage of our approach is that we can include mismatch probes which are especially suitable to identify aberrations, because they are less sensitive to gene expression variations.

Moreover, in *any *such model of expression estimation the residuals of the entire probe set containing a faulty probe is likely to be affected, so that errors are spread across the probe set and hence over the image; if one probe in a probe set is an outlier, e.g., very bright, all other probes would be slightly dark ghost images, similar to the 'ghosting' seen in Figure 4. Utilizing topological information for identification and elimination of blemishes has the advantage that suspect probes are identified *before *background correction, normalization and summarization take place. Thus, faulty data will not confound the preprocessing steps and further statistical analysis.

With the next generation of Affymetrix chips, the relevance of correcting for blemishes will even increase. Here, we analyzed U95 chips with 16 probe pairs per probe set. To make room for more probe sets, the number of pairs per set has been reduced to (as few as) 11 on the U133 chips. This, however, not only increases the standard error by 20%, and, thus the effect of any artefacts on the results, but also reduces the ability of model based methods to draw on probe set information. The number of neighbouring cells on a microarray, in contrast, is not adversely affected by reducing the size of the probe sets. In fact, smaller probe sets make it less likely that probe pairs from the same set are in close vicinity.

## Conclusion

We have presented an extremely simple method for finding blemishes on microarrays. The method's simplicity makes it robust and it does not rely on estimating model parameters. It sensitively tagged blemishes on chips that had passed our Gene Array Resource Center's quality control mechanism. Only one blemish (Figure 5) could have been readily seen in the raw images. That we found clear evidence of physical blemishes in the raw images for most of the artefacts identified on the pseudo images attests to the validity of the findings.

We have applied our method to an experimental dataset and were able to identify anomalies of different type. Approximately 25% of our chips are blemished, often more than once, and blemishes can cover areas from a few dozen to hundreds of probes. We examined the potential impact these blemishes have on the experiments. Failure to remove the blemishes from further analysis can materially affect the detection of subtle changes in experiments testing similar conditions. When applied to the Spike-in data set, the proposed method had an overall better sensitivity/(1-specificity) ratio.

For the future we propose to develop pattern recognition algorithms to automatically find and mask out suspected blemishes, and to modify the extant background correction and summarization algorithms to be able to properly handle missing data from blemish removal.

## Methods

Let **X**^(*i*)^, *i *= 1, ..., *n*, represent the intensity values of the *i*-th of *n *chips, each consisting of *m *× *m *(e.g., 650 × 650) cells . Assuming that biological systems respond to relative, rather than absolute differences in gene expression, for each pair of chips a matrix of pointwise (log) ratios is defined as



Given that the intensity at each cell is highly determined by the sequence of the probe [8], the spatial distribution of differences in log-intensities should have no identifiable features, except for probes belonging to probe sets related to the genes that are differentially expressed under the conditions the samples were taken. Here, we assume that the proportion of differentially expressed genes is small. Thus, since probes belonging to a probe set are (more or less) randomly distributed across the chip, cells of related genes are rarely located next to each other, so that no obvious pattern should be discernable. If, however, chip **X**^(*i*) ^has a localized 'defect', this should result in a similar pattern across all **R**^(*i*,*i*'≠*i*) ^in the region of the defect. To allow for visual inspection of such pattern, we draw on the fact that the distribution of differences in log-intensities should be (more or less) symmetrical, except for outliers caused by rare events affecting small areas in particular chips. Probe-wise outliers (due to both differential expression and defects) can be identified by comparing each chip to a measure of central tendency derived from all other chips. Although other measures of central tendency will be discussed below, we start our discussion with the special case of the arithmetic mean, which is known to be optimal in the classical linear model ([21])



Let **R**^(*i*,*i*') ^= Δ^(*i*,*i*') ^+ **D**^(*i*) ^- **D**^(*i*') ^+ ε where Δ^(*i*,*i*') ^indicates the random contribution from the differentially expressed genes, **D**^(*i*) ^describes the defects of the *i*-th chip, and ε other random errors. Then, **D**^(*i*) ^contributes not only to  (bars indicating the average over the index replaced by dot), but also, albeit with only 1/*n *of the intensity, to each of the other  as a 'negative shadow' or 'ghost' image. As the number of chips *n *increases, however, the law of large numbers allows for approximating the linear equation system (1), with hats indicating estimators, as



From (2), we get the linear equation system:



where **I **= (δ_*j *= *j*'_)_*j*,*j*' = 1...*n *_and **J **= (1)_*j*,*j*' = 1...*n*_. A system  has the trivial solution **Y **= **D **whenever column sums are zero (**JY **= **0**). As (2) guarantees that , setting  yields the solution



as the linear model estimate for the deviation of the *i*-th chip from the other chips. As the number of chips increases, ghosting reduces, so that any discernable pattern in  in the limit would suggest a defect.

The above justification for obtaining residuals within the linear model by subtracting the average is well known. Still, spelling out and justifying the individual steps above helps in two ways. First, we can fine tune the method for the particular situation we are faced with and, second, we can provide numerical examples comparing the proposed non-parametric with the traditional parametric approach. The justification for the choice of the arithmetic mean (average) as the measure of central tendency in linear models relies either on the law of large numbers and the central limit theorem or on the assumption that the distribution of errors is symmetrical, in general, and Gaussian, in particular. Neither assumption is easily justified for the errors caused by defects on a chip.

The arithmetic mean is known to be relatively sensitive to outliers. Thus, to discriminate outliers from observations close to the centre of the non-outliers, one would need either a very large number of chips or a measure of central tendency that is less likely to be affected by the outliers themselves. While microarray 'experiments' now typically consist of more than a single chip, the number of chips analyzed under comparable conditions is still too small to rely on the central limit theorem for outlier detection. With the number of chips in the single digits, even 'Winsorization' may not be feasible. Moreover, the need for choosing some Winsorization cut-off points adds an undesirable level of arbitrariness to the results. The median, as the most robust form of Winsorization, provides for a simple alternative measure of central tendency:
